# The Direct and Potentiating Antimicrobial Activities of Zafirlukast

**DOI:** 10.3390/antibiotics15090914

**Published:** 2026-09-16

**Authors:** Ed Siegwart, Ajay Mistry, John George

**Affiliations:** 1 Oppilotech Ltd., Stevenage SG1 2FX, UK; 2 School of Health, Leeds Beckett University, Leeds LS1 3HE, UK

**Keywords:** antimicrobial resistance, teichoic acids, potentiators, Zafirlukast

## Abstract

**Background**: Antimicrobial resistance is at alarming levels. Methicillin-resistant *Staphylococcus aureus* is a clinical concern and has been classified as one of the so-called ESKAPE pathogens. The increase in resistance coupled with the lack of new agents in development requires innovative ways of developing new antimicrobials. **Methods**: Here, we investigate an approved drug (Zafirlukast), which has been previously reported to have activity against *Mycobacterium*, for activities against other bacterial strains. Antibacterial activity was assessed using standard MIC testing along with checkerboard analysis to investigate potential synergies/antagonisms. In order to define the mechanism of action (MOA), a number of Zafirlukast-resistant mutant strains of MRSA (generated via serial passage) were sequenced. Biochemical analysis was performed on the target pathway to confirm the putative target of Zafirlukast in *Staphylococcus aureus*. **Results**: Zafirlukast showed modest direct-acting antimicrobial activity (0.5–32 μg/mL) against a panel of Gram-positive bacteria, including MRSA. It was also found that Zafirlukast synergised with a number of existing antibiotics. Remarkably, mutants forced to be resistant to Zafirlukast became resensitised to the β-lactams that they were originally resistant to. This is potentially a very useful property of a potentiator drug. Mutational analysis suggested the target of Zafirlukast in *Staphylococci* is LtaS (Lipoteichoic acid synthase), of which there are no currently approved inhibitors. Teichoic acid pathway involvement was further confirmed via biochemical analysis. **Conclusions**: Here, we establish that Zafirlukast has activities beyond *Mycobacterium* and *Gingivalis*, encompassing many Gram-positive bacteria including MRSA, and, for the first time, we report its putative target as LtaS. The repurposing of safe-to-use drugs is an attractive strategy to derisk drug discovery programmes. Zafirlukast has many attractive antimicrobial activities, which may encourage further investigations in this field. However, it should be noted that it may represent a safe starting scaffold, and further chemistry is likely to be needed for it to be used in the antimicrobial setting.

## 1. Introduction

Around 10 million deaths are expected to be attributed to infections caused by drug-resistant organisms by the year 2050 [1]. *Staphylococcus aureus* (*S. aureus*) is one of the pathogens that the WHO has placed in the ESKAPE group of pathogens (which are resistant organisms of serious concern). Around 60% of the population harbours transient colonisation by *S. aureus*, and 25–30% are permanently colonised [2], suggesting that a significant portion of the population is at some risk. The Centres for Disease Control and Prevention (CDCs) have listed the pathogen as an extreme threat, with approximately 100,000 deaths attributed to MRSA [1,3]. In addition to Methicillin, MRSA is also frequently resistant to other antibiotic classes, including newer agents such as Daptomycin and Linezolid [4,5]. This increase in resistance, coupled with the lack of new agents in pre-clinical or clinical development, has created the perfect storm, which seriously affects clinical treatment and management. Therefore, new innovative methods to detect and develop antimicrobials are much sought after.

Drug repurposing has long been a potential option, as using compounds that have already passed clinical trials and been deemed safe may help derisk the development process [6]. Approximately 30% of FDA approvals are made up of repurposed molecules, indicating that it is an attractive strategy for industry [7]. Although no repurposed molecules have been repositioned as antibiotics to date, several have shown promising in vitro activity [8,9]. One approved drug, Zafirlukast, has been identified has having antibacterial activity [10], capable of inhibiting the growth of *Mycobacterium tuberculosis*. Zafirlukast’s original clinical use is as a highly potent leukotriene antagonist for the treatment of asthma [11]. It is a complex synthetic molecule classified as a carbamate ester, an indole derivative and a N-sulfonylcarboxamide (see Pubchem 5717). Against *Mycobacterium*, its intracellular target is Lsr2, a nucleoid-associated protein (NAP) and recently DNA condensation has been observed in the presence of Zafirlukast, confirming and refining the MOA [12]. The asthma drug has also been reported to have activity against *Porphyromonas gingivalis* and *Streptococcus mutans*, although no mode of action has been identified as yet [13]. Importantly, Gram-positive bacteria do not possess Lsr2, so it is possible that Zafirlukast’s activity might extend to other Gram-positive bacteria, as molecules can frequently be pan-active.

Here, we investigate the broader antimicrobial properties of Zafirlukast to determine whether it is a promising candidate for repurposing. We also explore its potential mode of action in Gram-positive bacteria and investigate any synergies or antagonisms with other existing antibiotics.

## 2. Results

We determined the MIC value of Zafirlukast against a panel of clinically relevant bacteria (Table 1). Staphylococci, including *mecA*-containing and multidrug-resistant strains, possessed modest MICs of 8–16 μg/mL. The test Streptococci strains showed a wider range of MICs, from 0.5 μg/mL (*S dysgalactiae*) to 32 μg/mL for *S pneumoniae*, similar to the test L. monocytogenes and Bacilli strains. Importantly, a solvent-only control (1% DMSO) had no measurable antibacterial activity.

**Figure 1:**
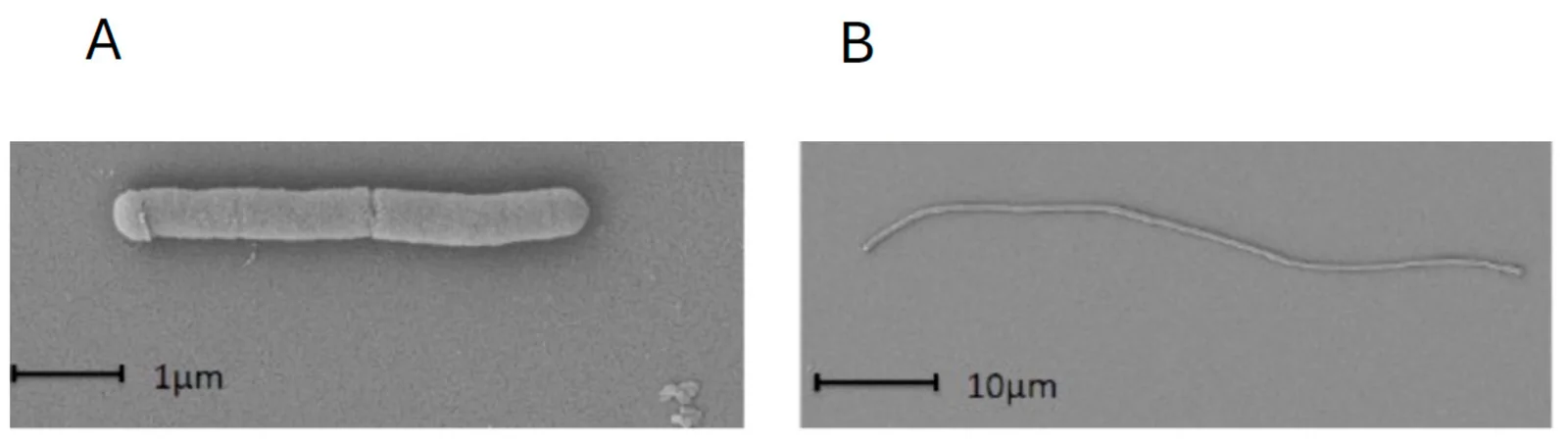
High-resolution scanning electron microscopy (SEM) image showing the surface morphology of *Bacillus subtilis* ATCC 6051 control cells (**A**) or (**B**) treated with 8 μg/mL Zafirlukast for 1 h at 37 °C. Imaging was performed at an accelerating voltage of 3 kV. The scale bar represents [e.g., 1 or 10 µm]. Seven randomly selected fields were chosen each containing 30–50 individual cells. Approximately 68% of cells exhibited the above morphological change.

We next examined the impact, if any, that Zafirlukast has on cellular morphology as determined by SEM (Figure 1). Bacillus subtilis treated at half the MIC value for 1 h demonstrated a filamentous phenotype, reminiscent of beta-lactam treatment [14].

Due to the relatively modest MIC values obtained for Zafirlukast against our test panel, we next investigated whether it may be a useful combining agent. Therefore, we conducted synergy via the standard checkerboard method using a mini panel of old but well-studied antibiotics. Table 2 indicates that Zafirlukast demonstrated synergy with Methicillin and Cephalosporin C, whilst exhibiting additive or indifference when combined with Ampicillin.

**Figure 2:**
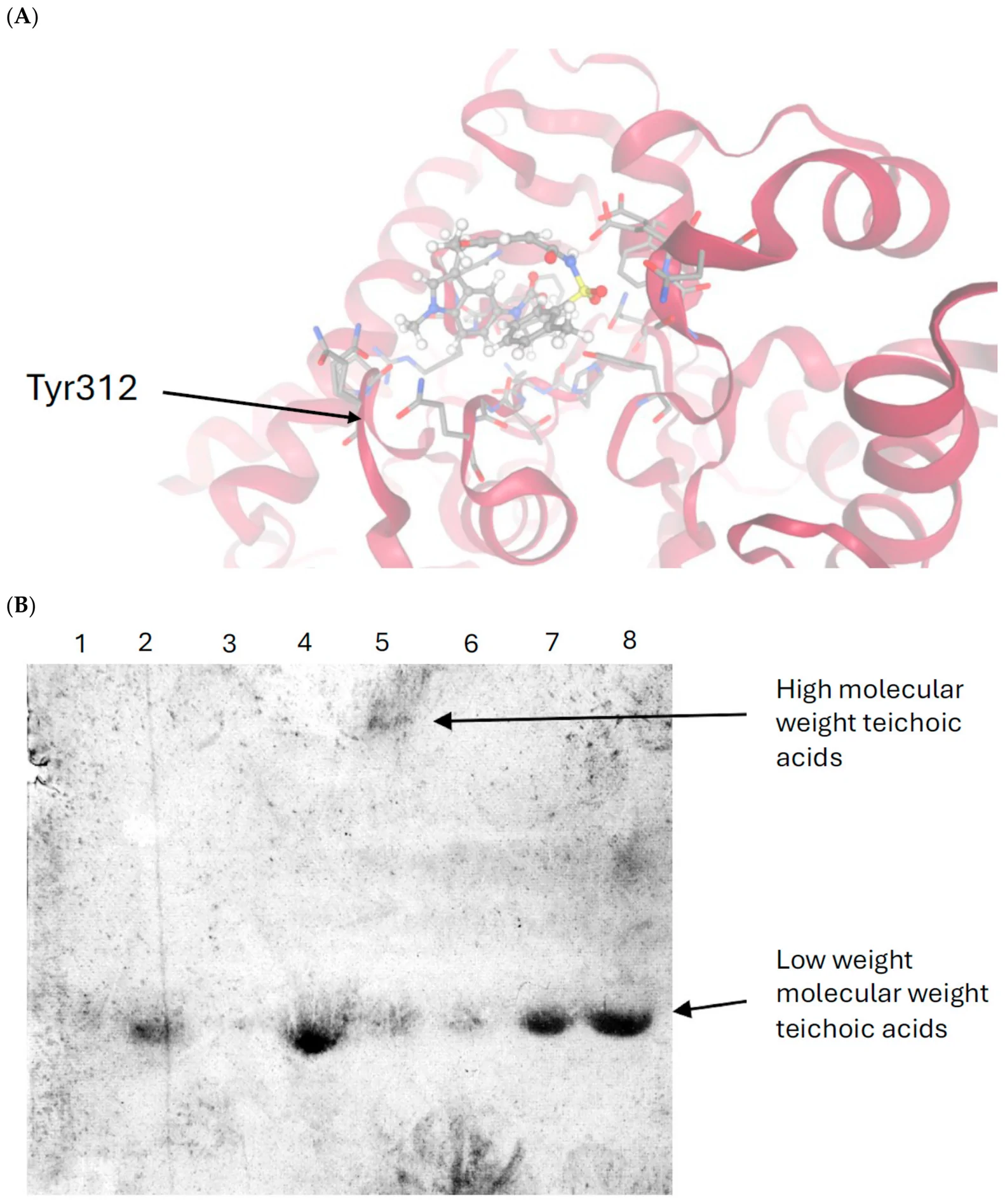
(**A**) Docking of Zafirlukast with LtaS (2W5Q-protein data bank) of *Staphylococcus aureus*. Tyrosine 312 is highlighted as one Zafirlukast-resistant mutant possessed a phenylalanine replacement at this site. Docking was carried out using Swiss Dock [17,18]. Parameters used Box centre 7-52-25; Box size 20-20-20, number of RIC = 1, cavity prioritisation = buried). (**B**) Teichoic acid gel. Each well contains 20 μg of purified teichoic acids from cells treated with 1–12 μg/mL of ZAF, 2–6 μg/mL ZAF, 3–3 μg/mL ZAF, 4–1.5 μg/mL ZAF. 5–represents teichoic acids from untreated cells. 6–1 μg/mL Tunicamycin, 7–0.5 μg/mL, 8–0.25 μg/mL Tunicamycin. Treatments were carried out for 3 h at 37 °C prior to teichoic acids extractions and purification.

**Figure 3:**
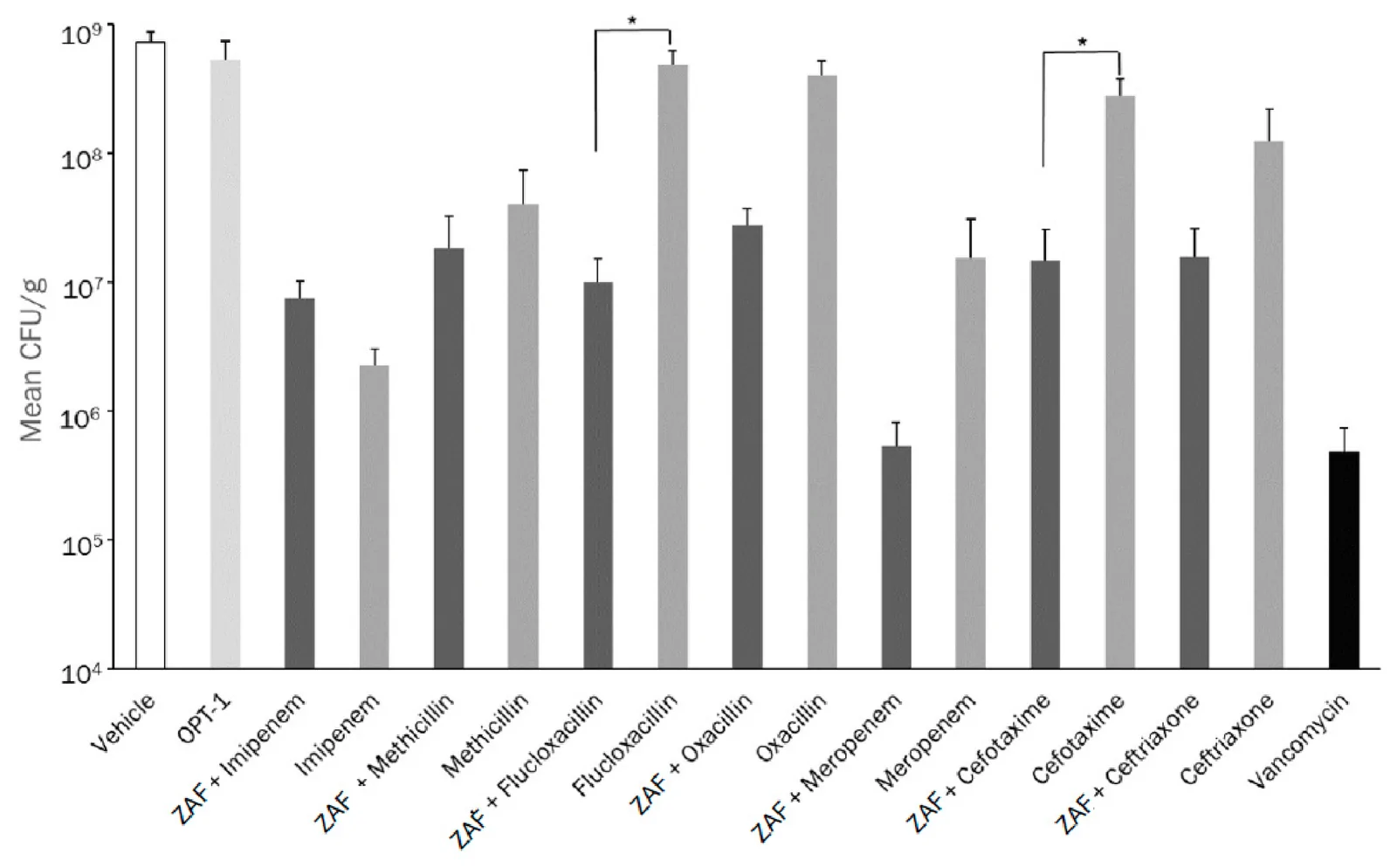
In vivo antibacterial activities of Zafirlukast (OPT-1) alone or in combination with a number of established antibacterial agents. Experiments were carried out in CD1 (neutropenic) mice. Zafirlukast (ZAF) was administered at 200 mg/kg and mice were infected with 10^6^ cfu of MRSA (OPP006). Mice were culled at 24 h and bacterial load in the kidneys was determined by cfu/mL (n = 5). Data presented are the arithmetic mean. A one-way ANOVA was applied to determine statistical significance, followed by Sidak’s multiple comparison test. * = *p* < 0.05.

To gain insight into the mode of action, we carried out serial passages in the presence of Zafirlukast to generate resistant mutants. This process tended to take 2–3 weeks in the presence of the compound until mutants with stable MIC shifts were obtained. It was also noted that the compound solubility suffered as the concentration was increased, creating a limit to the upper concentration achievable. However, 14 MRSA (EMRSA16-SSCmecType2 *mecA*) mutants were produced which possessed Zafirlukast MICs > 64 μg/mL, which represents a 4-fold increase from the parent. In order to define the mutants, we examined them for any cross-resistance to other antibacterial agents. Although we found no cross-resistance, we found that all mutants appeared to be resensitised to the antibiotics the parent was resistant to (Cefuroxime; Ceftriaxone; Ampicillin; Ceftazidime; Cefotaxime; and Piperacillin/tazobactam). Following passage in Zafirlukast-free media, MIC values for Methicillin and Oxacillin were determined. It was found that only six mutants maintained their resistance to Zafirlukast and simultaneously were resensitised to the β-lactams (Table 3). Sequencing revealed a number of mutations in *tarO*, the biochemical start point for teichoic acid synthesis. Many of these mutations resulted in the insertion of a premature stop codon or a truncated form of *tarO*, presumably inactivating the pathway in a similar manner to what has been observed for Targocil [15]. However, in one strain, a specific active site mutation was observed for the late-stage teichoic acid enzyme *ltaS*; here, Tyr312 was mutated to phenylalanine. Interestingly, for this mutant partial β-lactam potentiation was observed. Molecular docking using the crystal structure [16] (2W5Q PDB) revealed that Zafirlukast could interact with this enzyme and is predicted to interact close to Tyr 312 (Figure 2A). To confirm teichoic acid pathway involvement, we next analysed the impact of teichoic acid production in cells treated with Zafirlukast or Tunicamycin, a known teichoic acid inhibitor used as a positive control (Figure 2B). Finally, we assessed whether it is feasible for Zafirlukast to maintain its antimicrobial synergies in vivo using a standard murine model. Briefly, neutropenic CD-1 mice were infected with MRSA (IP) and treated with Zafirlukast alone or in combination with a number of clinically relevant antibiotics. Vancomycin was used as the gold standard treatment for MRSA in these experiments. Although no combination was as efficacious as Vancomycin, Zafirlukast showed significant synergies with Cefotaxime and Flucloxacillin (Figure 3), indicating that the in vitro activities carry through in vivo.

## 3. Discussion

Here we assessed whether the approved leukotriene antagonist Zafirlukast had wider antimicrobial activity than previously reported for Mycobacterium, *P. ginivalis* and *S. mutans* [10,12,13]. Despite lacking Lsr2, MIC testing revealed modest anti-Gram-positive activity (0.5–32 μg/mL; Table 1), which is generally higher than its growth inhibition of M. tuberculosis (1.5 μg/mL) [10,12]. However, the MICs obtained are unlikely to translate into clinical efficacy as a sole, direct-acting agent. Combining Zafirlukast with other existing antibiotics revealed synergies with some beta-lactam antibiotics (Table 2), suggesting its potential use as a combination agent. Although developing combination therapies is difficult because of the need to match PK and avoid undesirable DD interactions, there is precedent in the antimicrobial field; for example, endocarditis is often treated with multiple antibiotics.

Remarkably, mutants which were generated to be resistant to Zafirlukast exhibited re-sensitisation to the beta-lactams that the parent strain was resistant to. This could be a very useful property for an antibiotic adjuvant molecule and may hint at resistance-breaking opportunities. It may also indicate that Zafirlukast would be a useful potentiator molecule. Potentiators can enhance the activity of the combined agent [19,20]. For example, this can be achieved by reducing the activity of the resistance mechanism [21]—for example, blocking an efflux pump. However, there are likely many other means of potentiation, such as increasing cellular permeability or inhibiting a pathway on which resistance mechanisms depend.

Analysis of Zafirlukast-resistant mutants revealed several mutations in *tarO*, the first enzyme in the teichoic acid pathway. These mutations either truncated the enzyme or generated an early stop codon within the gene. This likely results in a non-functioning teichoic acid pathway and, therefore, any inhibition of components downstream of *tarO* would be rendered ineffective. Interestingly, teichoic acid biosynthesis in staphylococci is conditionally essential, meaning it can be switched off in vitro by inactivating early pathway enzymes. This mechanism has been observed previously for the teichoic acid inhibitors Targocil and Tunicamycin [15]. It remains to be seen whether this mechanism can operate in vivo. However, one Zafirlukast-resistant strain contained a mutation in the active site of Lipoteichoic acid synthase (LtaS) (Tyr312-Phe). Interestingly, this mutant only partially restored β-lactam sensitivity compared to the inactivation of the whole pathway. This is presumably because the mutation produces a less active enzyme, yielding some but limited teichoic acid levels; however, further work is required to confirm or refute this. Ltas in *S. aureus* is required for the polyglycerol phosphate backbone of LTA [22]. Interestingly, in its absence, bacteria demonstrate profound growth defects and are unable to grow unless the medium is modified to protect against osmotic shock [22,23]. Docking Zafirlukast with the crystal structure of LtaS revealed a potential binding site (Figure 3). It is not known whether this mutation reduced the activity of teichoic acid synthesis in this strain; however, it is likely, since it exhibited growth retardation. The binding pocket of LtaS has been extensively studied, and key residues include a substrate-binding loop comprising His347; Asp 349, Phe 353, Trp 354; Arg 356 and Leu384 [16], in addition to a catalytic nucleophile, Thr300. It can be seen that these residues are in close proximity to the predicted docking site and to the mutated residue in the resistant mutant. Biochemical analysis of teichoic acid synthesis under Zafirlukast-repressed conditions revealed a dramatic reduction in high-molecular-weight teichoic acid production. This was similar to what was observed for the control compound Tunicamycin (a known teichoic acid inhibitor) (Figure 2B). The reason repression of teichoic acids results in potentiation, or indeed re-sensitisation, is not fully elucidated. However, resistance PBPs such as MecA may be functionally dependent on these macromolecules, perhaps to spatially co-ordinate their activities [24]. In this context, the inhibition of early teichoic acid synthesis has been extensively studied; however, late-stage pathway components such as LtaS inhibition are much less studied, although some inhibitors of this enzyme have been reported [25]. None of these compounds have progressed, however, which may make Zafirlukast an attractive option.

Importantly, the potentiating activities of Zafirlukast were effective in vivo, significantly improving the efficacy of Cefotaxime and Flucloxacillin in a standard mouse model of infection (Figure 3). However, significant issues remain regarding the potential use of Zafirlukast in an antibiotic setting. Firstly, the concentration at which it is prescribed (20 mg) is far below what is required to achieve potentiation. This means that a clinical trial would still be required to assure safety at the newly indicated dose. Second, solubility issues may require further chemistry to reach the concentrations needed for clinical use.

## 4. Methods

### 4.1. Bacterial Strains, Media and Antimicrobials

The strains used in this study are the property of Oppilotech Ltd. (Stevenage, UK) unless otherwise stated. Strains were stored at −80 °C until required, at which point, purity plates were prepared by streaking on cation-adjusted Mueller–Hinton agar (CAMHBA) (Oxoid, Basingstoke, Hampshire, UK). Zafirlukast was resuspended in DMSO, before dilution in relevant media; the final concentration of DMSO did not exceed 1%. Solvent-only controls were checked prior to experimentation. Antibiotic discs used were from Sigma-Aldrich (Steinheim, Germany). All other antimicrobials (Imipenem, Methicillin, Flucloxacillin, Oxacillin, Meropenem, Cefotaxime, Ceftriaxone and Vancomycin) were prepared as concentrated stock solutions in sterile water.

### 4.2. MIC Testing

MICs were determined using the standard broth microdilution method (CLSI) in sterile 96-well microtitre plates. Each well contained a defined volume of broth (typically 50–100 μL) with decreasing concentrations of the antimicrobial agent arranged in a two-fold dilution series. Bacterial inocula were prepared by suspending colonies in sterile saline or broth to match a 0.5 McFarland turbidity standard. We further diluted the suspension in broth to achieve a final inoculum density of approximately 5 × 10^5^ CFU/mL in each well. Antimicrobial compounds were prepared as stock solutions in appropriate solvents based on their physicochemical properties and diluted in CAMHB or an equivalent growth medium. We performed serial two-fold dilutions to generate a concentration range spanning the expected inhibitory values. Plates were covered and incubated under aerobic conditions at 35–37 °C for 16–24 h. Following incubation, wells were assessed visually for bacterial growth. Growth was indicated by turbidity or pellet formation, whereas clear wells indicated inhibition. The MIC was defined as the lowest concentration of antimicrobial agent that completely inhibited visible bacterial growth. 

### 4.3. Synergy Testing

The interaction between Zafirlukast and other antimicrobial agents was evaluated using checkerboard methods, based on the CLSI broth microdilution methods above. Briefly, the first test compound is prepared in a doubling dilution concentration range running horizontally on a microtitre plate. This is overlaid with a second compound with a concentration range running vertically on the same plate and is then inoculated with the test organism to achieve a test inoculum of approximately 5 × 10^5^ CFU/mL. Plates are incubated, and the MIC of both individual drugs alone is ascertained (MIC_X_ and MIC_Y_), as well as all inhibitory concentration combinations of the two compounds together. These are then used to calculate fractional inhibitory concentration indices (FICIs) and mean FICI, which are interpreted based on scores from Odds et al. [26].

Calculate FIC_X_ and the FIC_Y_ for each inhibitory concentration:

$${\mathrm{FIC}}_{X}\;=\;\frac{{\mathrm{MIC}}_{X}\;\mathrm{combination}}{{\mathrm{MIC}}_{X}}$$

$${\mathrm{FIC}}_{Y}=\frac{{\mathrm{MIC}}_{Y}\;\mathrm{combination}}{{\mathrm{MIC}}_{Y}}$$

(1) $$\mathrm{FICI}\;=\;{\mathrm{FIC}}_{X}\;+\;{\mathrm{FIC}}_{Y}$$

### 4.4. Mutant Generation

CAMHB (containing ¼ MIC value of Zafirlukast) was inoculated with a single colony of MecA-containing *S. aureus* (315). After overnight incubation, if growth was apparent, 50 μL was used to inoculate CAMHB containing ½ MIC of Zafirlukast and re-incubated. Zafirlukast concentration was doubled on each occasion until cultures capable of growth in 4× MIC were produced. At this point, purity plates were prepared, and individual colonies were tested for stable and consistent increases in MIC value.

### 4.5. Next-Generation Sequencing and Informatics

Mutants were grown in CAMHB overnight before centrifugation at 10,000× *g* for 4 min to pellet the cells. Cells were washed 3 times in sterile ice-cold PBS before being snap frozen. Cells were then sent to Eurofins (Ebersberg, Germany) for sequencing. Sequencing data was stored on CLIMB (Aston University, Birmingham, UK) for analysis. For alignment, the *mecA*-containing *S. aureus* N315 was used as the backbone. We determined sequence differences using Minimap2 [27].

### 4.6. In Vivo Efficacy Tests

KWS Biotest (Bristol, UK) carried out all in vivo tests. CD-1 mice were randomly allocated to experimental groups (n = 5) and allowed to acclimatise for a week. Mice were rendered neutropenic by intraperitoneal (IP) injection with cyclophosphamide on day −4 (150 mg/kg) and day −1 (100 mg/kg). On day 0, MRSA COL was prepared to a suitable concentration (2 × 10^4^) IP in 3% hog mucin for IP infection. Zafirlukast dose was 200 mg/kg; Imipenem 10 mg/kg; Methicillin 35 mg/kg; Flucloxacillin 35 mg/kg; Oxacillin 35 mg/kg; Meropenem 35 mg/kg; Cefotaxime 35 mg/kg; Ceftriaxone 35 mg/kg and Vancomycin 35 mg/kg. All agents were delivered subcutaneously. After 24 h, mice were culled, and the cfu/mL present in the kidneys were determined by standard methods (n = 5). Statistical analysis was carried out using a one-way ANOVA.

### 4.7. Scanning Electron Microscopy

All SEM work was carried out at the Sir William Dunn School of Pathology (University of Oxford, Oxford, UK) using a JEOL JSM-6390 (Tokyo, Japan). Bacterial cells were prepared for scanning electron microscopy using a standard fixation and dehydration protocol. Briefly, bacterial cultures were harvested by centrifugation and washed in phosphate-buffered saline (PBS) to remove residual media. The cell pellet was fixed in 2.5% (*v*/*v*) glutaraldehyde prepared in 0.1 M sodium cacodylate buffer (pH 7.2–7.4) for 30–60 min at 4 °C to preserve cellular ultrastructure. Following primary fixation, samples were rinsed in buffer and post-fixed in 1% osmium tetroxide for 30–60 min to stabilise membrane lipids. The fixed cells were then dehydrated through a graded ethanol series (50%, 70%, 95%, and 100%), with increasing solvent concentrations to gently remove water and minimise structural damage. Dehydrated samples were dried using critical point drying (CO_2_) or chemical drying with hexamethyldisilazane (HMDS) to prevent artefacts associated with surface tension during evaporation. Dried specimens were mounted onto aluminium SEM stubs using conductive carbon tape and sputter-coated with a thin layer (~1–10 nm) of gold to enhance surface conductivity and prevent charging during imaging.

### 4.8. Cell Wall Teichoic Acid Analysis

Wall teichoic acid (WTA) was extracted from *Staphylococcus aureus* (COL) using a modified Fischer/Meredith procedure [28,29]. Briefly, purified cell wall sacculi were prepared by boiling in SDS and digesting with Proteinase K. WTA was released by treatment with 5% trichloroacetic acid at 60 °C for 4 h. Extracted polymers were separated on 20% native polyacrylamide gels and visualised by silver staining according to the method of Wolters et al. [30], with minor modifications.

## 5. Conclusions

In conclusion, we have found that the pre-approved drug Zafirlukast has antimicrobial activities beyond what has already been reported. The direct-acting activities are modest; however, it can potentiate and reactivate classes of antimicrobials to which bacteria are resistant. This is an extremely useful property that may extend the life of existing but now disused safe antimicrobials. Further chemistry will be required to fix some of the undesirable properties of Zafirlukast, but as the scaffold has been demonstrated to be safe to use, it may derisk future development.

## Figures and Tables

**Table 1:** MIC values for Zafirlukast against a number of important bacterial isolates. Values shown are the most frequent obtained following triplicate readings. MICs were carried out using the standard microbroth dilution method.

Strain	Isolate	MIC mg/L
*S. aureus*	ATCC 29213	16
*S. aureus*	MRSA clinical isolate	16
*S. aureus*	MRSA MDR clinical isolate	8
*S. aureus*	MU50 MRSA-VISA	8
*S. aureus*	EMRSA3-SSCmecType 1(MecA)	8
*S. aureus*	EMRSA16-SSCmecType2 (MecA)	8
*S. aureus*	EMRSA1-SSCmec Type3 (MecA)	8
*S. aureus*	EMRSA15-SSCmec Type4 (MecA)	8
*S. aureus*	MRSA Mup R	8
*S. pneumoniae*	ATC 49619	32
*S. pyogenes*	MLS macrolide resistance clinical isolate	8
*S. dysgalactiae*	Macrolide R clinical strain	0.5
*S. dysgalactiae*	MDR clinical strain	16
*S. pyogenes*	Susceptible control strain	8
*L. monocytogenes*	Antibiotic susceptible clinical strain	32
*L. monocytogenes*	Other clinical strain	8
*B. sub* *ti* *lis*	ATCC 6051	16
*B. cereus*	ATCC 14579	8
*E. faecalis*	VRE 102	16
*E. faecalis*	VRE569	16

**Table 2:** Fractional inhibitory values obtained using the standard checkerboard method for Zafirlukast combined with 4 beta-lactam antibiotics.

Combining Agent	FIC Value
Cephalasporin C	0.27, 0.4, 0.38
Methicillin	0.34, 0.62, 0.44
Ampicillin	1.12, 0.92, 1.32
Oxacillin	0.66, 0.82, 1.12

**Table 3:** Mutants obtained following serial passage in Zafirlukast resulting in a stable and reproducible shift in MIC values for Methicillin and Oxacillin. Fourteen mutants were obtained; however, only 6 were stable following passaging in Zafirlukast-free media. * represents a stop codon. MIC values represent the most frequent value from triplicate experiments.

Strain	Methicillin MIC μg/mL	Oxacillin MIC μg/mL	Zafirlukast MIC μg/mL	Mutation (Gene and Codon)	Proposed Mechanism
Parent	8	8	8	None	None
Mutant 001	1	1	32	*tarO* Q21 *	Inactivation of teichoic acid pathway.
Mutant 002	1	2	64	*tarO* W113 *	Inactivation of teichoic acid pathway.
Mutant 003	1	1	64	*tarO* Q154 *	Inactivation of teichoic acid pathway.
Mutant 004	0.5	1	32	*tarO* C460T	Protein truncation.
Mutant 005	1	1	>64	*secY* K425A	Unknown
Mutant 006	4	4	>64	*Ltas* T312P	Active site mutation.

## Data Availability

All the data for this project are presented within this report. All reasonable requests will be granted.
